# Surviving mothers and lost babies – burden of stillbirths and neonatal deaths among women with maternal near miss in eastern Ethiopia: a prospective cohort study

**DOI:** 10.7189/jogh.10.010413

**Published:** 2020-06

**Authors:** Abera Kenay Tura, Sicco Scherjon, Jos van Roosmalen, Joost Zwart, Jelle Stekelenburg, Thomas van den Akker

**Affiliations:** 1 School of Nursing and Midwifery, College of Health and Medical Sciences, Haramaya University, Harar, Ethiopia; 2 Department of Obstetrics and Gynecology, University Medical Centre Groningen, University of Groningen, Groningen, the Netherlands; 3 Department of Obstetrics and Gynecology, Leiden University Medical Centre, Leiden, the Netherlands; 4 Athena Institute, Vrije Universiteit Amsterdam, the Netherlands; 5 Department of Obstetrics and Gynecology, Deventer Ziekenhuis, Deventer, the Netherlands; 6 Department of Health Sciences, Global Health, University Medical Centre Groningen, University of Groningen, Groningen, the Netherlands; 7 Department of Obstetrics and Gynecology, Leeuwarden Medical Centre, Leeuwarden, the Netherlands

## Abstract

**Background:**

Although maternal near miss (MNM) is often considered a ‘great save’ because the woman survived life-threatening complications, these complications may have resulted in loss of a child or severe neonatal morbidity. The objective of this study was to assess proportion of perinatal mortality (stillbirths and early neonatal deaths) in a cohort of women with MNM in eastern Ethiopia. In addition, we compared perinatal outcomes among women who fulfilled the World Health Organization (WHO) and the sub-Saharan African (SSA) MNM criteria.

**Methods:**

In a prospective cohort design, women with potentially life-threatening conditions (PLTC) (severe postpartum hemorrhage, severe pre-(eclampsia), sepsis/severe systemic infection, and ruptured uterus) were identified every day from January 1st, 2016, to April 30th, 2017, and followed until discharge in the two main hospitals in Harar, Ethiopia. Maternal and perinatal outcomes were collected using both sets of criteria. Numbers and proportions of stillbirths and early neonatal deaths were computed and compared.

**Results:**

Of 1054 women admitted with PTLC during the study period, 594 women fulfilled any of the MNM criteria. After excluding near misses related to abortion, ectopic pregnancy or among undelivered women, 465 women were included, in whom 149 (32%) perinatal deaths occurred: 132 (88.6%) stillbirths and 17 (11.4%) early neonatal deaths. In absolute numbers, the SSA criteria picked up more perinatal deaths compared to the WHO criteria, but the proportion of perinatal deaths was lower in SSA group compared to the WHO (149/465, 32% vs 62/100, 62%). Perinatal mortality was more likely among near misses with antepartum hemorrhage (adjusted odds ratio (aOR) = 4.81; 95% CI = 1.76-13.20), grand multiparous women (aOR = 4.31; 95% confidence interval CI = 1.23-15.25), and women fulfilling any of the WHO near miss criteria (aOR = 4.89; 95% CI = 2.17-10.99).

**Conclusion:**

WHO MNM criteria pick up fewer perinatal deaths, although perinatal mortality occurred in a larger proportion of women fulfilling the WHO MNM criteria compared to the SSA MNM criteria. As women with MNM have increased risk of perinatal deaths (in both definitions), a holistic care addressing the needs of the mother and baby should be considered in management of women with MNM.

Despite the fact that in Ethiopia between 1900 and 2015 a two-third reduction of under-five mortality as part of the Millennium Development Goals was achieved, this was not accomplished for neonatal mortality [1-3]. Perinatal mortality (fetal loss from 28 weeks of gestation onwards) and early neonatal mortality (within 7 days after birth) remained among the highest in sub-Saharan Africa [1-5].

Maternal near miss (MNM) is increasingly applied as an indicator of quality of obstetric care, putting women who barely survived severe complications of pregnancy into focus [6]. The World Health Organization (WHO) suggested MNM criteria that were not always deemed suitable for use in low-income settings in sub-Saharan Africa [7,8], following which adapted “sub-Saharan African (SSA) MNM criteria have been suggested in a recent Delphi consensus study [9].

Although it is clear that complications leading to MNM also contribute to adverse perinatal outcomes, the magnitude of stillbirths and early neonatal deaths among women with MNM is largely unknown in low-resource settings [6,10,11]. In theory, addressing factors that contribute to maternal complications could also reduce adverse perinatal outcomes, and it is therefore important to quantify the association between MNM and perinatal mortality [12-15]. Moreover, although MNM is often considered a ‘great save’, quantifying this association will bring attention to women who, having survived a life-threatening complication themselves, will nonetheless still have to deal with the loss of a child and other adverse maternal outcomes [16-22].

Stillbirth is a silent tragedy, and in general, compared to maternal mortality, audited less frequently in health facilities in low-resource countries [23-26]. It is also possible that audit of perinatal mortality is sometimes forgotten if a woman’s life that was in danger of a life- threatening complication, was saved while her baby’s life was lost. The objective of this study was to assess proportion of stillbirths and early neonatal deaths among a cohort of women with MNM in eastern Ethiopia. Additionally, this paper compares stillbirths and early neonatal deaths among cohorts of women with MNM according to the adapted sub-Saharan African and the original WHO MNM criteria. Finally, we aimed to assess factors associated with adverse perinatal outcomes among women with MNM.

## METHODS

### Study setting and period

This study was conducted in Hiwot Fana Specialized University Hospital and Jugel Hospital in Harar, Ethiopia, from January 1st, 2016, to April 30th, 2017, as part of a larger study on MNM and pregnancy-related mortality [27]. In brief, during this 16-month period, all women admitted with potentially life-threatening conditions, PLTC (severe postpartum hemorrhage, severe pre-eclampsia, eclampsia, sepsis/severe systemic infection, and ruptured uterus) were identified on a daily basis by trained research assistants and followed until discharge [28]. Upon discharge, women fulfilling the adapted SSA MNM criteria were included [9]. MNM was defined as any woman who had severe obstetric complications according to the SSA MNM criteria.

### Data collection

Data on sociodemographic characteristics (maternal age, booking for antenatal care, gravidity, parity, referral status), obstetric conditions (onset of labor, mode of delivery, gestational age at birth, number of fetus), obstetric complications (obstetric hemorrhage, hypertensive disorders, sepsis, anemia, admission to intensive care unit, and receiving of blood), perinatal characteristics (vital status at birth, birthweight, 5^th^ minute Apgar score, admission to the neonatal intensive care unit), and maternal and perinatal vital status at discharge were collected by trained research assistants.

### Data processing and analysis

All women admitted to the participating hospitals during the study period constituted the source population. The study population consisted of all women with PLTC. The inclusion criteria were fulfilling MNM according to the sub-Saharan Africa MNM criteria [9]. Women with near misses related to abortion, ectopic pregnancy or were discharged before delivery were excluded from the analysis. Data were entered using EpiData v3.1 (www.epidata.dk) and analyzed using Stata v.13 software (StataCorp Inc, College Station, TX, USA). The main outcome of study was perinatal mortality: stillbirths and early neonatal deaths. Independent variables consist of maternal conditions and complications, institutional factors (referral status, admission dates, booking for antenatal care), and fetal conditions (birthweight, admission to neonatal intensive care unit, Apgar score and number of fetus). Bivariate and multivariate logistic regression were used to determine factors associated with perinatal mortality. Variables with *P*-value ≤0.25 in the bivariate analysis were retained for multivariate analysis. Significance level was set at *P*-value of <0.05.

### Ethics

This study was conducted as part of a larger prospective study of severe maternal outcomes (maternal near miss and mortality) in eastern Ethiopia [27]. The study protocol was reviewed and approved by the Institutional Health Research Ethics Review Committee of College of Health and Medical Sciences, Haramaya University, Ethiopia (Ref N: C/A/R/D/01/1681/16).

## RESULTS

**Figure 1:**
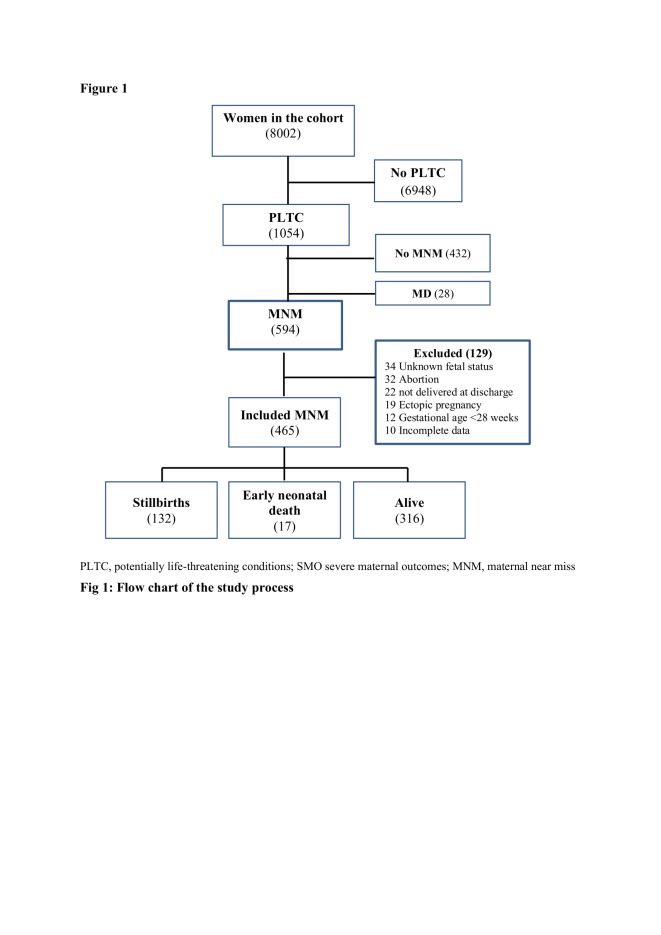
Flowchart describing the inclusion process in the study. PLTC – potentially life-threatening conditions, SMO – severe maternal outcomes, MNM – maternal near miss.

During the study period, of 8002 women admitted to both hospitals during pregnancy and childbirth, 7929 deliveries (including 7404 livebirths and 598 stillbirths) and 1054 women with PLTC were registered. The SSA MNM criteria identified 594 women with MNM while the WHO MNM criteria identified 128. After exclusion of women with near misses related to abortion, ectopic pregnancy, undelivered women, or unknown fetal status at discharge, 465 women with MNM were included in the analysis (**Figure 1**).

The mean age of women with MNM (n = 465) was 25.3 (±6.2) years ranging from 15-45. More than half (n = 282; 61%) were referred from other facilities and more than two-third (n = 316; 68%) were not booked for antenatal consultation. One hundred eighty-one (38.9%) women gave birth by cesarean section and hysterectomy was done in 44 (9.5%). With regard to complications, 231 (49.7%) had hypertensive disorders, 157 (33.8%) obstetric hemorrhage, and 109 (23.4%) sepsis. About 141 (30.3%) were anemic (Hb <7gm/dl) and 166 (35.7%) received at least one unit of blood (**Table 1**). A total of 149 perinatal deaths (132 stillbirths and 17 early neonatal deaths were registered), resulting in a stillbirth and early neonatal mortality rate of 284 and 36.6 per 1000 births respectively. The perinatal mortality rate was 320 per 1000 births in the SSA group and 620 per 1000 births in the WHO MNM group. The proportion of still births was 244 and 75 per 1000 births among women with PLTC and all women admitted to the hospital during the study period respectively. Thus, proportion of stillbirths per 1000 births was highest in the cohort of women with MNM (n=284).

**Table 1:** Sociodemographic characteristics of study participants with and without adverse perinatal outcomes

Variables		Adverse perinatal outcomes	
**Yes (n = 149), %**	**No (n = 316), %**
Maternal characteristics:			
Age (years)	<20	15(10.3)*	50 (15.8)
	20-35	104 (71.2)	232 (73.4)
	>35	27 (18.5)	34 (10.8)
Referred from other facility	No	47 (32.0)+	133(42.2)‡
	Yes	100(68.0)	182(57.8)
Received antenatal care:	Yes§	47 (31.5)	102 (32.3)
	No	102 (68.5)	214 (67.7)
Gravida	1	34 (23.1)2	137 (43.6)2
	≥2	113 (76.9)	177 (56.4)
Mode of delivery	Vaginal	79 (53.0)	205 (64.9)
	Cesarean‖	70 (47)	111 (35.1)
Obstetric complications:			
Obstetric hemorrhage	Yes	85 (57.0)	72 (22.8)
	No	64 (43.0)	244 (77.2)
Hypertensive disorders	Yes	47 (31.5)	184 (58.2)
	No	102 (68.5)	132 (41.8)
Sepsis	Yes	19 (12.8)	90 (28.5)
	No	130 (87.2)	226 (71.5)
Anemia	Yes	63 (42.3)	78 (24.7)
	No	86 (57.7)	238 (75.3)
Received blood	Yes	81 (54.4)	85 (26.9)
	No	68 (45.6)	231 (73.1)

*Missing information (n = 3).

+Missing information (n = 2).

‡Missing information (n = 1).

§At least one antenatal care.

‖Includes laparotomy.

Compared to the WHO MNM criteria, the SSA criteria picked up more perinatal deaths (149 vs 62) while the proportion of perinatal deaths was higher in the WHO group (62% vs 32%). Additionally, admission to neonatal intensive care unit, stillbirths and early neonatal deaths were more common among women in the WHO criteria compared to the SSA MNM group. Birthweight and 5^th^ minute Apgar score did not differ between the two groups (**Table 2**).

**Table 2:** Fetal outcomes among women with and without the WHO MNM criteria

Variables		Fulfilled WHO MNM criteria		Total	
Yes* (n = 100, %)	**No (n = 365, %)**	***P*-value†**
Fetal vital status at birth	Alive	42 (42.0)	291 (79.7)	333 (71.6)	**<0.001**
	Stillbirths	58 (58.0)	74 (20.3)	132 (28.4)	
Birth weight (grams)	<1500	8 (8.0)	19 (5.2)	27 (5.8)	0.476
	1500-2400	16 (16.0)	70 (19.2)	86 (18.5)	
	≥2500	76 (76.0)	276 (75.6)	352 (75.7)	
5^th^ minute Apgar score	<7	4 (4.0)	17 (4.7)	21 (4.5)	0.779
	≥7	96 (96.0)	348 (95.3)	444 (95.5)	
Admitted to neonatal intensive care unit	No	96 (96.0)	325 (89.0)	421 (90.5)	**0.035**
	Yes	4 (4.0)	40 (11.0)	44 (9.5)	
Neonatal outcome at discharge	Alive	38 (38.0)	278 (76.2)	316 (68.0)	**<0.001**
	Dead	62 (62.0)	87 (23.8)	149 (32.0)	

WHO – World Health Organization, MNM – maternal near miss

*Fulfil the sub-Saharan Africa criteria.

†χ2 test.

### Factors associated with perinatal mortality

Factors associated with perinatal mortality in the study participants (n = 465) are summarized in **Table 3**. Perinatal death was more likely among women who had antepartum hemorrhage (aOR 4.81; 95% CI = 1.76-13.20), grand multiparous women (aOR 4.31; 95% CI = 1.23-15.25), and women who fulfilled WHO MNM criteria (aOR 4.89; 95% CI = 2.17-10.99). No statistically significant association was observed between perinatal mortality and referral status, gestational age, and birthweight (**Table 3**).

**Table 3:** Factors associated with perinatal mortality among women with MNM in eastern Ethiopia

Variables		Perinatal deaths		
**No**	**cOR**	**aOR**
Ante partum hemorrhage	No	308	Ref	Ref
	Yes	157	**3.93 (1.77-8.74)**	**4.81 (1.76-13.20)**
Parity*	0	126	Ref	Ref
	1-4	208	0.93 (0.56-1.55)	
	≥5	127	**3.08 (1.81-5.24)**	**4.31 (1.23-15.25)**
Referred from other facilities†	No	180	Ref	
	Yes	282	**1.55 (1.03-2.35)**	
Gestational age	Pre-term Term(≥37weeks)	147	Ref	
		318	**0.45(.0.30-0.68)**	
Birthweight (gram)	≥2500	352	Ref	
	1500-2400	86	1.30 (0.79-2.15)	
	<1500	27	**8.94 (3.51-22.82)**	
Fulfilled WHO MNM criteria	No	365	Ref	Ref
	Yes	100	**5.21 (3.26-8.34)**	**4.89 (2.17-10.99)**

cOR – crude odds ratio, aOR – adjusted odds ratio, Ref – reference group, WHO – World Health Organization, MNM – maternal near miss

*Missing information (n = 4).

**†**Missing information (n = 3).

## DISCUSSION

Our findings indicate the high burden of adverse perinatal outcomes (stillbirths and neonatal deaths) among women with MNM. More than one in three of women with MNM went home with a dead baby. This highlights the importance of close monitoring of fetal conditions in those women. Adverse perinatal outcomes were more likely among grand multiparous women and women with antepartum hemorrhage. Given the fact that the WHO MNM tool is based on more severe clinical criteria identifying advanced disease with organ dysfunction, it is self-evident that perinatal outcomes in this group were worse (62 per 1000 births) compared to the SSA MNM group (32 per 1000 births).

The stillbirth rate of 284 per 1000 births is unacceptably high. This is almost twice as high as findings from a general population in public health facilities in southern Ethiopia (n=183) and more than ten times higher than the average stillbirth rate (25 to 31 per 1000 births) in the general population in low-income settings of southern Africa and Asia [29,30].

The stillbirth rate (284) in our study was found to be higher than in a cohort of women with MNM in Brazil (195 per 1000 births) [31]. The fact that stillbirths in our SSA MNM cohort were even higher than in the Brazilian cohort defined by the WHO MNM criteria shows the high burden of stillbirths in our setting, which may be related to differences in the quality of intrapartum care.

Although the proportion of stillbirths was higher (58%) among women fulfilling the WHO MNM criteria, the rate in the SSA group (28.4%) indicates that women with MNM according to SSA criteria are still in an advanced stage of disease. The common pathways to severe maternal and perinatal outcomes are potential areas of intervention for addressing the needs of both women and newborns [6]. Factors associated with adverse perinatal outcomes in this study (antepartum hemorrhage, lack of prenatal care) were comparable with findings from previous studies in Uganda [32], Nigeria [33], the Gambia [34], and Brazil [35]. We found that perinatal deaths were higher in absolute numbers among women in the SSA MNM group. This highlights the importance of considering the use of the SSA MNM criteria for more robust audit if better maternal and perinatal outcomes are to be achieved [27]. The strength of this study is the use of prospective case identification and large sample size. Some limitations should also be considered. First, this study was not population-based and MNM women with adverse perinatal outcomes who could not reach hospital are missed. Second, follow-up was limited to only seven days or until discharge. Some perinatal deaths and MNM events may have occurred after discharge.

## CONCLUSIONS

Behind the rescued life of a woman with MNM, often lies the silent tragedy of stillbirths and neonatal deaths [23-26]. Although these women survived the life-threatening complications, they have to be supported to deal with loss of baby since burden of stillbirths and early neonatal deaths is more common in women with MNM. Compared to the WHO MNM criteria, the SSA MNM criteria is a better choice for conducting robust perinatal audit for improving perinatal care and survival. The high stillbirth rate in this cohort highlights the importance conducting a thorough audit of perinatal deaths to identify opportunities to improve these outcomes in the future.
